# Modern Concepts in Regenerative Therapy for Ischemic Stroke: From Stem Cells for Promoting Angiogenesis to 3D-Bioprinted Scaffolds Customized via Carotid Shear Stress Analysis

**DOI:** 10.3390/ijms20102574

**Published:** 2019-05-25

**Authors:** Annabella Benedek, Daniel Cernica, Andras Mester, Diana Opincariu, Roxana Hodas, Ioana Rodean, Johanna Keri, Theodora Benedek

**Affiliations:** Clinic of Cardiology, Cardiac Critical Care Unit, University of Medicine and Pharmacy, Gheorghe Marinescu 38, 540139 Târgu Mureș, Romania; annabell.benedek@yahoo.com (A.B.); andras.mester@yahoo.com (A.M.); diana.opincardiu@yahoo.ro (D.O.); roxana.hodas@yahoo.ro (R.H.); ioana_patricia@yahoo.com (I.R.); johannakeri@gmail.com (J.K.); theodora.benedek@gmail.com (T.B.)

**Keywords:** ischemic stroke, endothelial shear stress, neuroprotection, neuroregeneration, 3D-bioprinted scaffolds, stem cells, regenerative therapy

## Abstract

Ischemic stroke is associated with a tremendous economic and societal burden, and only a few therapies are currently available for the treatment of this devastating disease. The main therapeutic approaches used nowadays for the treatment of ischemic brain injury aim to achieve reperfusion, neuroprotection and neurorecovery. Therapeutic angiogenesis also seems to represent a promising tool to improve the prognosis of cerebral ischemia. This review aims to present the modern concepts and the current status of regenerative therapy for ischemic stroke and discuss the main results of major clinical trials addressing the effectiveness of stem cell therapy for achieving neuroregeneration in ischemic stroke. At the same time, as a glimpse into the future, this article describes modern concepts for stroke prevention, such as the implantation of bioprinted scaffolds seeded with stem cells, whose 3D geometry is customized according to carotid shear stress.

## 1. Introduction

Ischemic stroke continues to represent a devastating disease, associated with a tremendous economic and societal burden. Despite major progress in the field of stroke prevention in recent years, only a few therapies are available at this moment for the treatment of ischemic brain injury, and their effectiveness is limited in a significant proportion of cases.

The main therapeutic strategies available at present for the treatment of ischemic stroke are reperfusion, neuroprotection and neurorecovery. Unfortunately, only a small percentage of patients with ischemic stroke can benefit from the timely initiation of acute reperfusion therapies. At the same time, the most potent neuroprotective drugs have been shown to be ineffective in reducing the volume of infarcted brain tissue. Therefore, strategies promoting neurorecovery have started to play a more significant role in the complex management of ischemic brain injury [1].

Results of recent preclinical trials have shown that an ischemic injury of the brain tissue promotes neurogenesis and angiogenesis [2]. Therefore, the manipulation of endogenous neural precursors and endothelial cells, in order to enrich the intrinsic potential of neural cell regeneration, could represent a potential therapy able to promote functional recovery after ischemic cerebral injuries [3].

Angiogenesis, as a complex physiologic endogenous process, has proved to be a key neurorestorative mechanism triggered by ischemic injuries, associated with improved functional outcomes after ischemic stroke. Although the current available data supporting the role of angiogenesis in ischemic stroke are mainly derived from experimental animal models, therapeutic angiogenesis and vasculogenesis also seem to represent a promising tool to improve the prognosis of cerebral ischemia in clinical settings [4,5,6].

This review aims to present the current status of regenerative therapy for ischemic stroke and also the main results of major clinical trials addressing the effectiveness of neuroregenerative therapies for ischemic stroke. At the same time, as a glimpse into the future, the article describes modern concepts for stroke prevention, such as the implantation of customized bioprinted scaffolds, designed on the basis of carotid shear stress analysis.

## 2. Regenerative Tools in Modern Neurology

Stem cells (SCs) are self-renewing and undifferentiated cells capable of differentiating into more than 200 types of cells. The potential therapeutic effect of SCs is related to their role in cell differentiation, inflammation, immunomodulation and the stimulation of endogenous repair processes, such as angiogenesis and neurogenesis [7,8,9]. SC therapy has emerged as an encouraging regenerative tool in modern medicine, with potential applications in ischemic stroke as well as in other neurodegenerative disorders [10,11]. Cell-based therapies derive from the ability of SCs to proliferate and differentiate into new cells able to replace the damaged tissue. In the case of ischemic brain lesions, the hope behind SC therapy is that the replacement of damaged brain tissue by new cells originating from SCs could lead to the regeneration of the brain [8,12].

SC therapies are mainly based on two types of approach—endogenous and exogenous, both with their own advantages and disadvantages [11,13]. Endogenous SC therapy is based on the capacity of these cells to activate without inducing an immune response. In the case of ischemic stroke, this is the preferred type of SC therapy as it contributes to spontaneous neurogenesis in response to brain injury, in parallel with a reduced rate of adverse events. The exogenous approach is, by definition, the transplantation of SCs into the patient. This represents a promising therapy because exogenous SCs can replace the brain cells injured by stroke and can stimulate the secretion of neurotrophic factors [13].

### 2.1. SCs with Potential Applications in the Treatment of Stroke

Recent studies have investigated multiple cell lines capable of differentiating into potentially mature donor neuronal cells for the treatment of stroke. The most promising SC types from this point of view are neural stem cells (NSCs), endothelial stem cells (ESCs), inducible pluripotent stem cells (iPSCs), bone marrow stem cells (BMSCs), multilineage-differentiating stress-enduring cells (Muse), mesenchymal stem cells (MSCs), dental pulp stem cells (DSCs), adipose-derived stem cells (ADSCs) and c-kit^+^ cells [10,12,14,15,16].

NSCs result from the embryonic or fetal central nervous system, and their use can lead to different outcomes in acute and chronic stroke. These cells are able to differentiate into neurons, astrocytes and oligodendrocytes [11,17]. The neurorecovery potential of endogenous NSCs represents an attractive therapeutic target of regenerative medicine. Although NSCs are found in specific neurogenic regions, only those derived from the subventricular zone have the ability to migrate to the ischemic area caused by stroke [18]. The main advantage of NSC therapy in stroke is their potential to ameliorate neuroinflammation and to provide blood–brain barrier (BBB) protection, functional neuronal recovery, neurogenesis and angiogenesis.

MSCs are non-hematopoietic multipotent cells situated in the perivascular regions of different tissues, capable of differentiating into adipocytes, chondrocytes, osteoblastic cells, neurons, glia or endothelial cells [18,19]. They act through various mechanisms, such as immunoregulation, angiogenesis or neurogenesis, and represent an outstanding source of microRNA, growth factors and exosomes [7].

BMSCs are non-hematopoietic cells situated in the bone marrow with various functions in the proliferation and differentiation of hematopoietic cells [14,20]. Recent studies proved that BMSCs are able to influence neural behavioral after stroke, playing a significant role in neural cells’ proliferation and healing process due to their “nursing effect” and anti-inflammatory action [11,14,21]. The neuroprotective effect of BMSCs is mediated by the secretion of neuroprotective factors and the reduction of glutamate secretion [14].

Muse cells represent endogenous non-tumorigenic cells able to adapt under stress conditions, integrate into various damaged tissues and differentiate into neural cells, including neurons [14,22].

ESCs are bone marrow-derived immature cells that participate in endothelial repair and hemostasis through the secretion of cytokine and growth factors. Studies on mice have shown a significant reduction in the infarcted area after ESC transplantation, through their multiple beneficial effects at the level of cerebral vessels, leading to increased capillary density and reduced apoptosis [23,24].

DSCs are self-regenerating SCs that can rapidly proliferate, possessing the ability to secrete anti-inflammatory cytokines and neurotrophic factors, thus maintaining neuronal survival and stimulating axon regeneration and neuronal function recovery [12].

A new addition to the known types of stem cell sources with therapeutic potential is adipose tissue. Stem cells isolated from white adipose tissue represent an extremely versatile class of stem cells, holding greater differentiation potential and multipotency compared to other sources of stem cells [25]. Furthermore, in a recent study, exosomes from modified ADSCs were able to promote functional recovery after stroke in a rat model by improving angiogenesis. In this study, the administration of ADSC-derived exosomes increased cell proliferation and decreased neuron cell death compared with the control [26]. At the same time, ADSCs demonstrated the capacity to stimulate the expression of neuroprotective interleukins and brain-derived neurotrophic factors in the acute phase of brain injury, thus ameliorating brain damage after intravenous administration [27].

### 2.2. Growth Factors for Therapeutic Angiogenesis after Ischemic Stroke

Vasculogenesis involves de novo vessel formation by bone marrow-derived endothelial progenitor cells (EPCs) [28]. In ischemic conditions, EPCs are mobilized from bone marrow to hypoxic tissue in order to stimulate the neovascularization process [29]. Experimental studies have shown that an increased number of EPCs circulating during the first few days after the acute ischemic event is associated with a better outcome [30,31,32,33]. Moreover, intravenous administration of BMSCs in animal ischemic models proved to enhance the angiogenesis process [34]. Therefore, the exogenous supplementation of EPCs in stroke subjects might be a promising potential therapeutic strategy [35].

Besides cell-based therapies, growth factors have also been demonstrated to be involved in angiogenesis-based recovery after ischemic stroke, with vascular endothelial growth factor (VEGF) as the prototypical pro-angiogenic mediator [36]. Optimistic results from monotherapies involving VEGF administration proved that the delivery of VEGF to ischemic tissue could promote neurorecovery either directly via its neuroprotective properties or indirectly by inducing the angiogenesis process [37,38,39]. Moreover, preclinical studies showed a direct link between the administration of VEGF, stimulation of angiogenesis and reduction of brain infarct volume, with further improvement in neurological deficits [40,41,42]. However, the effect of VEGF therapy proved to be critically affected by the dosage, route of administration and time of delivery in relation to stroke onset.

Gene transfer with VEGF increases vascular permeability, a process required for further new endothelial cell migration. However, under specific conditions, beside its beneficial role, VEGF may actually induce hemodynamic steal by overexpression. Further, an untitrated response may lead to an alteration of the BBB with subsequent brain edema, vasodilatation and aberrant systemic hemodynamics [41,43,44,45].

The beneficial effect of VEGF proved to be time-dependent in relation to the ischemic process onset. Early administration in the hyperacute phase may promote edema formation and the hemorrhagic transformation of ischemic lesions, while delayed administration may present positive neurorepair effects. In terms of the administration route, intra-arterial and intravenous administration may alter the BBB, while the direct cortical or intracerebroventricular application of VEGF proved to be associated with a beneficial neuroprotective response. However, recent results showed that the adverse vascular permeability effects of VEGF might be obviated by a concomitant administration of angiopoietins [46,47,48].

### 2.3. Modulation of the Endogenous Angiogenic Response after Ischemic Stroke

While the transplantation of exogenous NSCs involves the risks of cellular death, failed integration and formation of tumors, endogenous NSCs can be involved in the regeneration process after stroke without any risk. However, previous experimental studies proved the intrinsic potential of endogenous NSCs to be insufficient, therefore external stimuli might be required to exploit their full therapeutic potential [49].

It is well known that an early initiation of physical activity after the acute event is able to enhance the angiogenic response, with further improvement in long-term outcomes, after stroke [50]. In the recovery period, physical training is able to induce a more robust angiogenic capacity through an increased production and recruitment capacity of EPCs [51]. The magnitude of this increase is directly associated with a better functional outcome [33]. Moreover, exercise preconditioning was proven to increase the tolerance to brain ischemia via a series of mechanisms, including the induction of VEGF and the stimulation of angiogenesis [52].

Pharmacologic interventions on the endogenous angiogenic response could also play a significant role in the modulation of angiogenic response after stroke. In animal models of focal cerebral ischemia, statins show neuroprotective and neurorestorative effects, which are independent of the level of cholesterol. These effects result in a limitation of ischemic insult and an improvement in functional outcome. Data available from in vivo and ex vivo research studies show that statins at low doses induce a biphasic, dose-dependent effect, with proangiogenic actions via the upregulation of EPCs and the induction of the expression of angiogenic factors (such as VEGF), while at higher concentrations they induce a strikingly contrasting angiogenic response [53,54,55,56].

Recent studies demonstrated that type 5 phosphodiesterase inhibitors are an important modulator of angiogenic potential [57,58,59]. The administration of sildenafil or tadalafil in animal ischemic models can enhance angiogenesis and improve functional recovery when initiated in the hyperacute phase of the ischemic event [60,61]. At the same time, several experimental findings suggest a cytoprotective effect of glitazones via the upregulation of both the number and function of EPCs [62,63].

Neural cell adhesion molecule (NCAM)-derived peptide FG loop (FGL) has been proven to be involved in the natural modulation of synaptogenesis, neurogenesis and SC proliferation. Via its capacity to cross the BBB, the small synthetic FGL could represent an attractive candidate molecule for enhancing endogenous NSC regeneration properties. In cerebral ischemia conditions, previous animal in vivo studies showed a significantly increased endogenous NSC mobilization with further acceleration of the endogenous regeneration process after systemic FGL treatment. Moreover, anti-inflammatory effects of FGL administration have been postulated, as NCAM mimetic modulates the M1/M2 polarization of microglia with further protective effects. Thus, NCAM-FGL presents the potential to enhance the cerebral self-repair mechanism after the acute ischemic event [49].

## 3. Therapeutic Effects of SCs after Ischemic Stroke: Neuroprotection or Neuroregeneration?

The use of SCs has exhibited promising results in pre-clinical neurological injury models as well as in neurodegenerative diseases. Several animal models have been developed for ischemic stroke to investigate the underlying repair mechanism and the therapeutic potential of SCs. In healthy conditions, the BBB is impenetrable even for cells of the peripheral immune system. Ischemic conditions activate matrix metalloproteinases (MMPs), which cause the disruption of the BBB, allowing activated immune cells to penetrate into the brain tissue, followed by the release of inflammatory mediators causing edema, an increase in intracranial pressure, hypoxia and subsequent further cell death. These circumstances offer the opportunity for administered SCs to directly penetrate and infiltrate into the injured brain territory in order to exhibit their reparative effect [64].

Numerous studies have demonstrated the efficiency of SC transplantation in different phases of the post-stroke period. SCs administered in the early phases (0–2 days) of the event had better survival rates compared to later (6 weeks) transplantation. These studies concluded that the administration of SCs in the acute and sub-acute phases of ischemic stroke had a neuroprotective role, which was evidenced in a reduced infarct volume and improved functional recovery.

The reparative mechanism is achieved via the multiple ways in which SCs can mediate the regeneration process. The endogenous repair mechanism relies on the expression of neural progenitors in the subventricular and subgranular zone of the brain, where SCs can mediate the upregulation of chemotactic and angiogenic factors that ultimately lead to the formation of new blood vessels in the infarcted zone and favor the migration of neuroblasts to restore the affected neurons. SCs can also provide trophic support for the ischemic parenchyma by normalizing the microenvironment and improving the survival of neurons [65]. This process is achieved by the secretion of stimulating factors, such as brain-derived neurotrophic factor and glial cell-derived neurotrophic factor, which provide support for the damaged regions, prevent further cell death and additionally promote neurogenesis, leading to improved functional recovery [66].

## 4. Paracrine Effects of SCs and Neuroinflammation

Another mechanism by which SCs stimulate neuroregeneration is via autocrine and paracrine effects [67]. BMSCs have the ability to regulate the inflammatory response by secreting chemokines and growth factors, such as nerve growth factor, colony-stimulating growth factor and VEGF, which were proven to limit cell destruction and were associated with improved functional recovery following ischemic stroke in pre-clinical studies [68]. Chronic neuroinflammation mediated by astrocytes and microglia extends over the sub-acute phase of an ischemic stroke and can lead to cerebral edema, neuronal destructions and a further extent of the injured brain zone. The sub-acute administration of SCs can mediate and suppress oxidative stress and apoptosis while stimulating neural reorganization and neuroregenerative processes, such as angio-, neuro-, or synaptogenesis [65,69,70].

Inflammation plays an important role in the regenerative process of an injured tissue by moderating cell migration, adhesion and promoting angiogenesis [71]. Recent studies highlighted that the implanted SCs not only act as a replacement for the affected tissue but also exhibit an important paracrine effect. Induced pluripotent SCs, embryonic SCs and BMSCs can be genetically edited to express anti-inflammatory, antiapoptotic and neuroprotective proteins with the promotion of angiogenesis and infiltration of NSCs. Recent studies suggest that combinative therapies of SC transplantation and the administration of growth factors (e.g., granulocyte-CSF) can lead to better functional outcomes compared to monotherapy by promoting angiogenesis and neurogenesis, and by reducing neuroinflammation [72].

## 5. Therapeutic Stem Cells after Ischemic Stroke: Current Status

In patients with stroke, treatment options include a combination of thrombolysis and catheter-based interventional procedures in the acute phase, followed by the administration of neuroprotective pharmacological agents. Neuroprotective medications failed to demonstrate promising results with respect to patient recovery and neurological outcomes. Thrombolysis is restricted by a short timeframe applicability and several patient-related contraindications, while catheter-based interventional procedures require a specific infrastructure with trained specialists in comprehensive stroke centers [73,74,75]. Moreover, combining cell therapies with the current treatment options, as well as their use in subjects who are eligible for neither lytic nor interventional therapies (the “no-option” patients), could represent an alternative solution associated with an overall improvement in neurological outcomes, reduced adverse events and increased health-related quality of life [76].

Since 2005, there has been a significant increase in the publication of clinical trials regarding SC therapies in stroke patients, using a wide array of different cell types [77]. Preliminary results suggest that cell therapies for stroke are feasible, with acceptable safety regardless of the delivery route, cell type or administration time from index event [78].

A search in the National Institutes of Health clinical trial database using the keywords “stem cells” and “ischemic stroke” (www.clinicaltrials.gov) (access on 3 March 2019) identified a number of 12 clinical trials completed. The studies included a total of 761 ischemic stroke subjects who underwent SC therapies via various routes of administration (Table 1). The chosen delivery route included intravenous SC infusion in five trials, exclusive intraarterial administration in the ipsilateral middle cerebral artery in three studies, an intracerebral delivery route in three trials, and a mixed intraarterial–intravenous route in one study. All the 12 trials are phase 1 or 2 studies, which aimed to evaluate the safety and feasibility of SC therapies in these patients, with a follow-up period ranging from 7 days to 24 months.

The completed trials tested the efficacy and safety of cell administration in ischemic stroke, with a large intervention timeframe from the index event ranging from hours to years. However, the intracerebral administration is reserved for patients with chronic stroke, usually at 6 to 60 months from the index event, while the intraarterial and intravenous administrations are more suitable within hours or days of stroke onset. Unfortunately, each cell delivery route is associated with different complications. Intracerebral administration, usually performed by stereotactic neurosurgery at the site of the damaged tissue, is associated with several procedural complications, including intracerebral hemorrhage and tissue damage secondary to the introduction of delivery devices, such as cannulas or needles. Despite this, the translation of SCs in unifocal lesions has been proven safe and feasible, the benefits outweighing the potential risks of brain damage. Nonetheless, the risk–benefit balance is reversed in multifocal lesions or repeated brain injections. Other complications associated with this route of injection include headache, somnolence or subdural hematomas [75,91,92,93,94].

In order to avoid the procedural complications of the stereotactic intracerebral implantation of SCs, alternative administration routes have been proposed, including intraventricular and intrathecal deliveries as well as intranasal catheter infusion, which is still undergoing preclinical animal investigation, with promising results in cerebral malignancies [95,96,97,98].

The intravascular transplantation of SCs, including intravenous and intraarterial routes, is a less invasive procedure that has been tested in clinical trials for ischemic stroke patients. Studies have shown that intravenous and intraarterial SC transplantation is associated with a significant decrease in the neurological deficits of stroke patients and a reduced size of the cerebral infarction and can even initiate brain tissue regeneration by releasing various cytokines and growth factors [99,100,101,102,103]. However, both intravenous and intraarterial routes seem to be associated with an array of complications and risks [75]. These include the formation of cellular emboli with consecutive systemic and pulmonary vascular obstruction, microembolism within the cerebral microvasculature, the capillary trapping of cells with impaired function due to the loss of concentration of cellular product at the lesion site as well as immunological response against the cell graft, which typically occurs in non-autologous transplantation.

Such complications can be avoided by using autologous cells for the prevention of immunological reactions, and by using cells with a smaller size and diameter, such as bone marrow-derived mononuclear cells. These types of cells are less likely to be filtered, in comparison to NSCs or MSCs [104,105,106,107,108,109].

Other complications that are not related to the delivery method of SCs in stroke patients include tumor and teratoma development, uncontrolled tissue proliferation and the ectopic implantation of the cell graft [75,110,111,112]. Seizures are a frequent adverse event following stroke, and their occurrence has also been linked to cell therapies for non-neurological conditions. [113,114]. Interestingly, Rosado de Castro et al. (2013) have shown that intraarterial implantation is associated with a lower seizure rate compared to the intravenous administration of SCs in a population of subacute ischemic stroke patients [78].

The success of cell therapies for stroke also depends on the type of cell used, which may vary. Embryonic SCs have the capacity to evolve in different neuronal and glial cellular elements, thus restoring the synaptic connections in ischemic brain lesions [115,116]. Another cellular lineage used in ischemic stroke is represented by the neural precursor cells that physiologically reside in the hippocampus and are able to form interneuronal connections, but also differentiate into neurons and astrocytes [76,117,118]. MSCs can be harvested from different histological sites, including bone marrow, adipose tissue, placenta and umbilical cord, and are able to differentiate into virtually any type of tissue. Bone marrow-derived mesenchymal autologous stem cells (BMMSCs) were the subject of many clinical and preclinical studies on ischemic stroke as they have been shown to endorse synaptogenesis and brain tissue regeneration and to improve motor and sensory functions, concomitantly having immunomodulatory effects on the inflammatory mediated immune response following cerebral ischemia. Autologous BMMSCs have the advantage of being safe from triggering autoimmune reactions, which may be present in the use of non-autologous cell therapies, and of having been proven safe and feasible in human clinical trials for stroke [119,120,121,122]. Induced pluripotent SCs, derived from programming fibroblasts and mononuclear cells from the peripheral blood, have been successfully used in stroke patients. Their use has been associated with absent immune rejection, reduced brain infarct zone, the recovery of neurological functions, neuroprotection by preserving the normal brain metabolism and diminishing the deleterious inflammatory response triggered by cerebral ischemia [123,124,125].

ADSCs have also demonstrated a significant potential to produce neurological improvement after stroke. In a recent study, allogeneic adipose-derived mesenchymal SCs produced neurological improvement in a rat model of stroke, via stimulating both angiogenesis and neurogenesis [126]. At the same time, another recent study demonstrated the potential of ADSCs to improve rehabilitation and functional recovery in experimental stroke [127].

Six ongoing active studies were identified in the search in the National Institutes of Health clinical trial database (www.clinicaltrials.gov) (accessed on 03 March 2019), out of which two are phase I interventional studies, two are phase II interventional randomized double-blind trials, one is a phase II/III open-label randomized study and one is a randomized double-blind placebo-controlled study on cell translation in ischemic stroke at different timeframes from the onset of the acute cerebrovascular event (Table 2). The evaluated route of delivery for SCs in the six studies is either intracerebral, via stereotactic neurosurgical interventions, or by the intravenous administration of the cell product, with none of the ongoing trials using the intraarterial route. The time from the index event to the study intervention varies from a minimum of 36 h up to 90 months, with an overall estimated stroke population of 816 subjects and a follow-up period ranging from 3 to 12 months. 

The primary outcome measures are the improvement of functional and structural neurological functions evaluated by various scales, including the modified Rankin Scale, the Fugl–Meyer motor scale or the National Institutes of Health Stroke Scale (NIHSS) [132,133,134].

Investigation of Neural Stem Cells in Ischemic Stroke (PISCES III) is another ongoing Phase IIB randomized, placebo-controlled clinical trial which uses stereotactic intracerebral injections of NSCs in ischemic stroke patients 1 year after the event, with an assessment of the functional improvement of the patient at 6 months’ follow-up.

The results of these ongoing studies will contribute to elucidating the role of SC therapy in ischemic stroke and to identifying the most appropriate timing and route of administration in order to maximize their neuroprotective and neuroregenerative effects.

## 6. Carotid Endothelial Shear Stress and Ischemic Stroke

One of the major causes of ischemic stroke is the embolization of an atheromatous material, most frequently originating from an atherosclerotic plaque located in a carotid artery. Atheromatous plaque formation, progression and vulnerabilization is a complex process influenced by various factors, one of them being represented by endothelial shear stress (ESS) [135,136,137,138].

ESS is also influenced by atherosclerotic plaque morphology. Unstable atherosclerotic plaques generally present a typical phenotype, which includes a more voluminous necrotic core, higher lipidic burden, spotty calcifications within the plaque as well as a lower calcium density compared to non-vulnerable plaques. Additionally, one of the possible culprit factors for atheromatous plaque vulnerabilization is the modification of ESS [139].

ESS represents the friction forces acting on the endothelium as a result of blood flow and has been demonstrated to play a crucial role in atherosclerosis [140]. It has been suggested that ESS regulates the production of endothelium-derived vasoactive factors, maintaining vascular tone and protecting the vascular wall from atherosclerosis [141]. A low shear stress at the level of carotid arteries has previously been associated with a risk of plaque progression and also with an increased carotid intimal-media thickness, a surrogate marker of atherosclerosis [142,143,144].

The alteration of the carotid ESS plays a significant role not only in atheromatous plaque formation and progression but also in its vulnerabilization, a process that modifies the composition of the plaque towards a more unstable phenotype, increasing the risk of distal embolization and thus being directly linked with the risk of stroke. In a clinical study on 22 stroke patients and 143 controls, Jeong et al. demonstrated that carotid ESS is significantly lower in patients who experienced ischemic stroke as compared to controls, indicating a possible causal relationship between carotid ESS and ischemic brain injury [145].

Preclinical studies have demonstrated that low ESS and oscillatory ESS can cause thin-cap fibro-atheroma formation, a marker of increased vulnerability in atherosclerotic plaques, and may facilitate pro-inflammatory macrophage programming at this level [146]. A recent study on 100 patients undergoing endarterectomy found that the alteration of carotid ESS is directly associated with embolic signals during carotid exposure in endarterectomy, demonstrating the role of ESS in atheromatous plaque embolism, a main cause of ischemic stroke [147]. Moreover, the relationship between low ESS and ischemic stroke has been clearly evidenced by the study of Jeong et al., who found that carotid ESS is significantly lower in patients with lacunar infarction as compared to controls [145]. At the same time, a recent study published by Pedigri et al. on ApoE^−/−^ mice, demonstrated a direct link between the magnitude and direction of shear stress and the composition of atherosclerotic plaques in the carotid arteries. Authors showed that ESS magnitude contributes to the transformation of plaque morphology into a more vulnerable phenotype, while variations in ESS magnitude and direction may promote plaque formation with more stable features [148]. This study indicates that the modification of ESS via external intervention may lead to a modification of plaque morphology and decrease the risk of plaque embolization and stroke. However, the nature of such an intervention is still uncertain, and this approach remains a real challenge.

A potential intervention for the regulation of carotid ESS, changing it into a more physiologic pattern, could be the implantation of dedicated scaffolds at the level of carotid artery stenosis, scaffolds with a 3D geometry customized on the basis of personalized ESS analysis by computational image processing.

## 7. Three-Dimensional Bioprinted Scaffolds Seeded with Stem Cells in Carotid Arteries: An Emerging Tool for Stroke Prevention

It is well known that spontaneous cellular regeneration following ischemic stroke is not sufficient for the functional restoration of neural tissue [149]. Engineered scaffolds can provide a unique biocompatible three-dimensional microenvironment that can endorse the growth and in vivo survival of therapeutic cells seeded on the scaffolds. Thus, the transfer of various SCs has been assessed to establish their potency and safety in the treatment of stroke. The SC types most used for bioprinting applications are NSCs, MSCs, HSCs, iPSCs and ESCs [123].

It has been shown that MSCs can replicate the biological three-dimensional network of cells and the extracellular matrix, resulting in MSCs with an “in vivo-like” microenvironment, which can be better preserved [150,151]. Moreover, recent studies have shown that MSCs can be assembled as spheroid-shaped cellular structures by 3D engineering techniques providing efficient differentiation toward neuronal-like phenotypes [152].

It has also been shown that the neuroprotective properties of NSCs may represent fundamental characteristics of their biological constitution [153]. An electrically conductive three-dimensional scaffold was recently designed as an innovative NSC delivery system that improved the recovery of neurological functions after stroke [154]. Current applications of NSCs impregnated on an engineered scaffold and transplanted into the adult rat spinal cord determined promising effects by improving the reduction of necrosis in the brain parenchyma and by preventing inflammation and glial scar formation [155]. The improvement of dynamic culture parameters through bioprinting could aid the efficient construction of 3D SC-seeded scaffolds [156,157].

## 8. Tissue Engineering and Bioprinting for Ischemic Stroke

Advances in regenerative medicine are increasingly offering new frontiers to repair damaged tissue by using biomaterials. Thus, engineered tissues are designed to encompass specialized regeneration characteristics for different tissues or organs.

Specific regenerative tissues can be generated by using bioprinting, a modern technology which consists of mixing a number of cells and biomaterials distributed with a high precision in order to obtain compositional characteristics of host tissues [158]. Various techniques can be used for bioprinting, such as: Laser-based printing using a laser to vaporize a site in the scaffold layer where bioink drops, droplet-based printing by delivering small droplets of bioink sequentially to form tissues, extrusion-based printing by extruding bioink layer-by-layer and stereolithography-based printing using a light projector to selectively deliver bioinks plane-by-plane [159].

The bioprinting process starts with designing scaffold geometry on the basis of clinical images, which are converted to a stereolithography (STL) file. The selected cell types and biomaterials form the bioink, which is bioprinted onto the scaffold by depositing the bioink under the control of a computer. The engineered tissues are verified by microscopy, and the bioprinted scaffolds are then moved to an incubator for culturing [160,161]. The most frequently used cells to fabricate this kind of highly specialized tissue are BMSCs, MSCs and SCs from amniotic fluid or placenta [162].

The efficacy of tissue engineering used in ischemic stroke depends on a few generic conditions, such as: Using three-dimensional scaffolds to achieve biocompatible integration in stroke tissue, using an appropriate quantity of cells to initiate the regeneration process and the employment of adequate growth factors that control cell differentiation [163].

### Advanced Biomaterials in the Treatment of Stroke

Biomaterials are compounds used in tissue engineering designed to encapsulate drugs or molecules. In stroke treatment, the main advantage of using biomaterials is the possibility to enhance graft cell survival, proliferation, migration and differentiation, favoring integration within the host neuronal tissue, with minimal immune response [163,164,165]. Biomaterials can be either natural or synthetic, offering different properties, which can influence biomaterial effectiveness and host tissue response [166].

Among natural polymers, hyaluronic acid, alginate and collagen are the most frequently used compounds that have already been tested and used in medical applications, including in the treatment of stroke [166,167]. Hyaluronic acid is used as an injectable hydrogel, applied in order to gain a controlled transfer of erythropoietin (EPO) in the stroke treatment, which endorses neurogenesis and tissue repair [168]. Alginate is another compound derived from brown algae. It has been especially used as an encapsulating biomaterial of VEGF, which induces structural and functional protection from ischemic stroke damage [169]. Collagen is an elemental component of the extracellular matrix, which provides appropriate structural and immunogenic properties. It has been used as a hyaluronan–heparin–collagen hydrogel mixture and as collagen type I.

Synthetic biomaterials are engineered compounds with analogous structural characteristics to surrounding cells, allowing for better extracellular networking and biochemical stability. Poly L-lactic acid-co-glycolic acid is a synthetic biomaterial used especially for three-dimensional scaffolds in tissue engineering and as a vehicle for delivering a number of therapeutic factors, such as T3 thyroid hormone, EGF and EPO. The combination of these factors has been shown to determine improved neurogenesis and promote the development of a local vascular network [170,171]

A series of recent clinical studies evaluated the clinical safety of engineered tissues seeded with SCs [172,173]. One clinical study investigated the effect of such an approach based on autologous MSCs administered several months after stroke using MRI control, which detected a decrease of 20% in lesion volume with no adverse events [119]. In another clinical study, HSC therapy for acute stroke led to improved clinical outcomes as expressed by a significant reduction in the National Institute of Health Stroke Scale score at 6 months after the stroke event [100]. Preliminary data indicate that biomaterials sustaining tissue engineering and facilitating SC grafting in the damaged brain could represent a promising technique for stroke treatment. The clinical safety and biocompatibility of engineered tissues are still the main issues to be solved in order to introduce this approach in clinical settings.

## 9. Bioprinted Scaffolds Customized on the Basis of Carotid Shear Stress Analysis: A Modern Approach for Stroke Prevention

Atherosclerotic plaque formation occurs more frequently in areas with low or oscillatory ESS, such as bifurcations or inner arterial curvatures, anatomical conditions which favor non-laminar flow [140,174]. This observation led to the hypothesis that the correction of anatomical conditions that favor oscillatory ESS, leading to the restoration of a more physiological ESS pattern, could be associated with a more stable phenotype of atherosclerotic plaques in the entire carotid tree, thus reducing the risk of atheromatous plaque embolization into the brain arteries and therefore reducing the consequent risk of stroke.

This correction could be achieved by the implantation of customized scaffolds at the site of severely stenotic carotid arteries, scaffolds with a 3D geometry preliminarily tested via a computerized simulation of carotid flow. Computational fluid dynamics based on complex image post-processing have become largely available in recent years, allowing flow simulation at different levels in the arterial tree [175]. This is a modern technology largely used nowadays to virtually test implantable devices via determining the pattern of arterial flow in response to the implantation of various biomaterials. Therefore, this technique allows the selection of the most appropriate implantable biomaterial type, configuration or size, namely the one which is associated with the most physiological pattern of ESS in virtual experiments.

**Figure 1:**
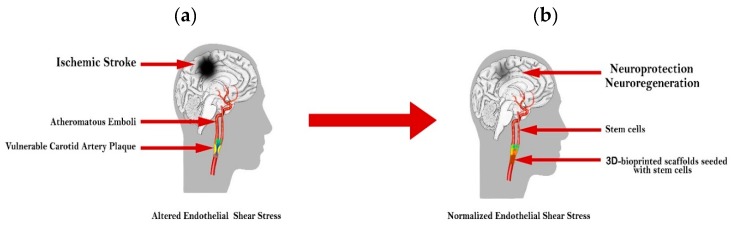
Stem cell therapy in ischemic stroke: (**a**) Vulnerable carotid artery plaque with altered endothelial shear stress (ESS) resulting in ischemic stroke; (**b**) 3D scaffold providing plaque stabilization, the correction of carotid stenosis and ESS with simultaneous neuroprotection and neuroregeneration at the level of the ischemic brain.

Taking into consideration the benefits demonstrated by SC therapy in ischemic stroke and its potential for neuroprotection and neuroregeneration, a modern therapeutic approach could combine the production of customized 3D scaffolds for the correction of carotid stenoses with the possibility to bioprint SCs onto these scaffolds. The concept of this modern approach is illustrated in Figure 1.

This revolutionary therapy would integrate in the same platform the beneficial effects resulting from the correction of carotid ESS with the therapeutic potential of SCs, providing plaque stabilization at the level of carotid arteries with simultaneous neuroprotection and neuroregeneration at the level of the ischemic brain. This requires the integration of multiple cutting-edge technologies, such as computational ESS analysis, scaffold customization, advanced 3D bioprinting and modern tissue engineering techniques.

At present, this remains a challenging hypothesis that should be tested not only in virtual experiments but also in clinical settings, representing a modern approach for stroke prevention that requires further validation.

## 10. Conclusions

In conclusion, regenerative therapy represents a key component in the complex management of ischemic stroke, and the main results of major clinical trials indicate beneficial effects of SCs in ischemic brain injury, facilitating angiogenesis, neuroregeneration and neuroprotection. New therapies are still under investigation in this clinical setting, including ones that modulate ESS for stroke prevention or the use of bioprinted scaffolds for brain regeneration. As a glimpse into the future, a challenging approach based on the development of bioprinted scaffolds seeded with SCs customized on the basis of carotid shear stress analysis remains to be further validated.

## Figures and Tables

**Table 1:** Completed clinical trials on stem cell research in ischemic stroke patients in the clinicaltrials.gov database.

Year	Title	Study Design	Objectives	Number of Enrolled Patients	Condition	Stem Cell Type	Route of Administration/Delivery	Time after Stroke	Follow-up Period	Primary Outcome Measures	Clinical Trial Identification Number
2015–2017	**Intraarterial Stem Cells in Subacute Ischemic Stroke [79]**	Phase 1; interventional; prospective randomized end observer blinded study; test group (stem cell therapy) vs. control group (standard of care)	Evaluation of the safety of intervention	229 (18–80 years old)	Acute MCA ischemic stroke	Autologous BMMNC stem cells	Intraarterial: Ipsilateral MCA	0–15 days	6 months	- Change in NIHSS - Symptomatic intracranial hemorrhage - New ischemic lesion - Death	NCT03080571
2009–2011	**Stem Cell Therapy for Acute Ischemic Stroke Patients [80]**	Phase 2; interventional; prospective randomized end observer blinded study; test group (stem cell therapy) vs. control group (standard of care)	Evaluation of the efficacy of intervention	120 (18–70 years old)	MCA/ACA ischemic stroke	Autologous BMSCs	Intravenous	7–29 days	6 months	Functional ability to perform activities of daily living on MBI score	NCT02425670
2014–2017	**A Phase II Efficacy Study of Intracerebral CTX0E03 DP in Patients with Stable Paresis of the Arm Following an Ischemic Stroke [81]**	Phase 2; interventional; prospective randomized efficacy study; non-comparative study	Evaluation of the efficacy of intervention	23 (≥40 years old)	MCA ischemic stroke; arm paresis	Allogenic human neural stem cell (CTX DP)	Intracerebral	>28 days	12 months	A minimum of 2 points of improvement in the ARAT test number 2	NCT02117635
2014–2017	**Reparative Therapy in Acute Ischemic Stroke with Allogenic Mesenchymal Stem Cells from Adipose Tissue, Safety Assessment, a Randomized, Double-Blind Placebo-Controlled Single-Center Pilot Clinical Trial (AMASCIS-01) [82]**	Phase 2; interventional; prospective randomized double-blind, efficacy study; test group (stem cell therapy) vs. control group (placebo)	Evaluation of the efficacy of intervention	19 (60–80 years old)	Ischemic stroke	Allogenic MSC from adipose tissue	Intravenous	12 h	24 months	- Serious adverse events - Neurological and systemic complications - Development of tumors.	NCT01678534
2008–2011	**Intravenous Autologous Bone Marrow-Derived Stem Cells Therapy for Patients with Acute Ischemic Stroke [83]**	Phase 2; interventional; prospective randomized double-blind, safety, feasibility and efficacy study; test group (stem cell therapy) vs. control group (placebo)	Evaluation of the safety, feasibility and efficacy of intervention	120 (18–70 years old)	Acute ischemic stroke	Autologous BMSCs	Intravenous	7–30 days	6 months	Difference between the two groups in MBI score	NCT01501773
2011–2015	**Study to Examine the Effects of MultiStem in Ischemic Stroke (MASTERS) [84]**	Phase 2; interventional; prospective randomized double-blind, safety and effectiveness study; test group (stem cell therapy) vs. control group (placebo)	Evaluation of the safety and potential effectiveness of intervention	134 (18–83 years old)	Ischemic stroke	Adult stem cell investigational product, MultiStem (BMSCs, allogenic)	Intravenous	1–2 days	7 days 90 days 365 days	- Frequency of dose-limiting adverse events - Stroke recovery	NCT01436487
2011–2015	**A Phase 1/2A Study of the Safety and Efficacy of Modified Stromal Cells (SB623) in Patients with Stable Ischemic Stroke [85]**	Phase 1, phase 2; interventional; safety and efficacy study; single group assignment	Evaluation of the safety and potential effectiveness of intervention	18 (18–75 years old)	Chronic, stable ischemic stroke patients	Modified stem cells, SB623	Stereotactic surgical implantation	6–60 months	2 years	Safety (WHO criteria); Improvement in stroke symptoms	NCT01287936
2009–2010	**Efficacy Study of CD34 Stem Cell in Chronic Stroke Patients [86]**	Phase 2; interventional; prospective randomized double-blind, safety and effectiveness study; test group (stem cell therapy) vs. control group (conventional treatment)	Evaluation of the safety and efficacy of intervention	30 (35–70 years old)	MCA ischemic stroke	Autologous peripheral blood stem cell (CD34+)	Intracerebral	6–60 months	1,2,4,12 weeks, 6,12 months	NIH-stroke scale (NIHSS)	NCT00950521
2010–2017	**Intravenous Stem Cells After Ischemic Stroke** **[87]**	Phase 2; Interventional; prospective randomized double-blind, safety and effectiveness study; test group (stem cell therapy) vs. control group (conventional treatment)	Evaluation of the safety and efficacy of intervention	31 (18–70 years old)	Ischemic stroke	Autologous MSCs	Intravenous	14 days	2 weeks, 1,2,4,6 months; 1,2 years	Feasibility and tolerance of the intravenous injection of autologous mesenchymal stem cells in patients with carotid ischemic stroke	NCT00875654
2008–2011	**Autologous Bone Marrow Stem Cells in Middle Cerebral Artery Acute Stroke Treatment [88]**	Phase 1, phase 2 trial. Interventional; prospective.	Evaluation of the safety and efficacy of intervention	20 (18–80 years)	MCA * ischemic stroke	Autologous CD34+ BMSCs	Intraarterial—in the MCA *	5–9 days	1,3,6 months	Absence of new neurological deficits and adverse effects during the timeframe	NCT00761982
2007–2012	**A Phase I/II Safety and Tolerability Study Following the Autologous Infusion of Immuno-selected CD34+ Subset Bone Marrow Stem Cells into Patients with Acute Total Anterior Circulation Ischemic Stroke [89]**	Phase 1, phase 2 trial. Interventional; prospective; non-randomized; single group study; safety and tolerability study.	Evaluation of the safety and tolerability of intervention	5 (30–80 years old)	Total or partial anterior circulation syndrome; acute ischemic stroke	Autologous CD34+ BMSCs	Intraarterial—in the ipsilateral MCA *	7 days	6 months	Adverse events graded according to CTC toxicity criteria and laboratory test results	NCT00535197
2005–2011	**Study of Autologous Stem Cell Transplantation for Patients with Ischemic Stroke [90]**	Phase 1, phase 2 trial. Interventional; prospective; non-randomized; single group study; safety and feasibility study	Evaluation of the safety and feasibility of intervention	12 (18–75 years old)	MCA * ischemic stroke	Autologous BMSCs	Intraarterial Intravenous	3 h to 90 days	4 months	Absence of new neurological deficits during the procedure and at follow-up	NCT00473057

* MCA—middle cerebral artery; ACA—Anterior cerebral artery; NIHSS—National Institutes of Health Stroke Scale; BMMNC—Bone marrow-derived mononuclear cell; BMSCs—Bone marrow-derived stem cells; MBI—Modified Barthel Index; MSC—mesenchymal stem cells.

**Table 2:** Ongoing registered clinical trials for various deliveries of stem cells in patients with ischemic stroke.

Brief Title	Study of Modified Stem Cells (SB623) in Patients with Chronic Motor Deficit from Ischemic Stroke [128]	Investigation of Neural Stem Cells in Ischemic Stroke (PISCES III) [129]	Intracerebral Transplantation of Neural Stem Cells for the Treatment of Ischemic Stroke [130]	Treatment Evaluation of Acute Stroke for Using in Regenerative Cell Elements (TREASURE) [131]	Pilot Investigation of Stem Cells in Stroke (PISCES) [8]	MultiStem^®^ Administration for Stroke Treatment and Enhanced Recovery Study (MASTERS-2) [84]
**Clinical trial identification number**	**NCT02448641**	**NCT03629275**	**NCT03296618**	**NCT02961504**	**NCT01151124**	**NCT03545607**
**Eligible patients**	Ischemic stroke (aged 18–75 years)	Ischemic stroke in the supratentorial region (aged 35–75 years)	Chronic motor ischemic stroke (aged 35–60 years)	Acute ischemic stroke (aged over 20 years)	Ischemic stroke involving subcortical white matter or basal ganglia (aged 60–85 years)	Ischemic stroke involving the cerebral cortex (aged over 18 years)
**Time from index event**	6–90 months	6–12 months	3–24 months	36 h	6 months to 5 years	18–36 h
**Intervention**	Stereotactic, intracranial injection of SB623 cells vs. sham surgery	Stereotactic surgery with an intracerebral injection of 20 million CTX0E03 Drug Product stem cells in the adjacent stroke area vs. sham surgery	One-time stereotactic, intracranial injection of a hNSC line, NSI-566 (neural stem cells) vs. sham surgery	Intravenous administration of 1.2 billion HLCM051 cells vs. placebo	Surgical delivery of CTX0E03 neural stem cells in the damaged cerebral area	Single intravenous infusion of MultiStem^®^ 18–36 h after stroke vs. placebo
**Study type**	Phase 2; interventional; double-blind, randomized	Phase 2; interventional; placebo-controlled, randomized	Phase 1; interventional; single group assignment	Phase 2, 3; interventional; open-label, randomized	Phase 1; interventional; single group assignment	Phase 3; interventional; double-blind, placebo-controlled, randomized
**Estimated enrollment number**	156 subjects	110 subjects	18 subjects	220 subjects	12 subjects	300 subjects
**Primary outcome measure**	Improvement of FMMS * by ≥10 points	Improvement of Mrs * by ≥1 point	Adverse events	Proportion of subjects with an excellent outcome defined by the functional assessments **	Incidence of adverse events **	Shift analysis for mRS *
**Time frame for primary outcome measure**	6 months	6 months	24 months	90 days	12 months	90 days
**Start date**	January 2016	August 2018	June 2012	November 2017	June 2010	July 2018
**Estimated completion date (to final enrollment)**	May 2019	November 2019	May 2018	March 2020	March 2023	December 2020

* FMMS—Fugl–Meyer Motor scale; mRS—Modified Rankin Scale; MSCs—mesenchymal stem cells; ** <Excellent outcome> is defined as an mRS score of ≤1 (scale, 0 to 6), a NIHSS score of ≤1 (scale, 0 to 42), and a BI score of ≥95 (scale, 0 to 100).

## References

[1] Amemori T., Romanyuk N., Jendelova P., Herynek V., Turnovcova K., Prochazka P., Kapkalova M., Cocks G., Price J., Sykova E. (2013). Human conditionally immortalized neural stem cells improve locomotor function after spinal cord injury in the rat. Stem Cell Res. Ther..

[2] Xiong Y., Mahmood A., Chopp M. (2010). Angiogenesis, neurogenesis and brain recovery of function following injury. Curr. Opin. Investig. Drugs.

[3] Zhang Z.G., Chopp M. (2009). Neurorestorative therapies for stroke: Underlying mechanisms and translation to the clinic. Lancet Neurol..

[4] Liu X.S., Zhang Z.G., Zhang R.L., Gregg S., Morris D.C., Wang Y., Chopp M. (2007). Stroke induces gene profile changes associated with neurogenesis and angiogenesis in adult subventricular zone progenitor cells. J. Cereb. Blood Flow Metab..

[5] Hayashi T., Noshita N., Sugawara T., Chan P.H. (2003). Temporal profile of angiogenesis and expression of related genes in the brain after ischemia. J. Cereb. Blood Flow Metab..

[6] Krupinski J., Kaluza J., Kumar P., Kumar S., Wang J.M. (1994). Role of angiogenesis in patients with cerebral ischemic stroke. Stroke.

[7] Bhartiya D. (2019). Clinical Translation of Stem Cells for Regenerative Medicine: A Comprehensive Analysis. Circ. Res..

[8] Kalladka D., Sinden J., Pollock K., Haig C., McLean J., Smith W., McConnachie A., Santosh C., Bath P.M., Dunn L. (2016). Human neural stem cells in patients with chronic ischaemic stroke (PISCES): A phase 1, first-in-man study. Lancet.

[9] Abbasi-Kangevari M., Ghamari S.H., Safaeinejad F., Bahrami S., Niknejad H. (2019). Potential Therapeutic Features of Human Amniotic Mesenchymal Stem Cells in Multiple Sclerosis: Immunomodulation, Inflammation Suppression, Angiogenesis Promotion, Oxidative Stress Inhibition, Neurogenesis Induction, MMPs Regulation, and Remyelination Stimulation. Front. Immunol..

[10] Yu S.P., Wei Z., Wei L. (2013). Preconditioning strategy in stem cell transplantation therapy. Transl. Stroke Res..

[11] Thwaites J.W., Reebye V., Mintz P., Levicar N., Habib N. (2012). Cellular replacement and regenerative medicine therapies in ischemic stroke. Regen. Med..

[12] Mead B., Logan A., Berry M., Leadbeater W., Scheven B.A. (2017). Concise review: Dental pulp stem cells: A novel cell therapy for retinal and central nervous system repair. Stem Cells.

[13] Koh S.H., Park H.H. (2017). Neurogenesis in stroke recovery. Transl. Stroke Res..

[14] Kuroda S., Koh M., Hori E., Hayakawa Y., Akai T. (2018). Muse Cell: A New Paradigm for Cell Therapy and Regenerative Homeostasis in Ischemic Stroke. Adv. Exp. Med. Biol..

[15] Baker E.W., Platt S.R., Lau V.W., Grace H.E., Holmes S.P., Wang L., Duberstein K.J., Howerth E.W., Kinder H.A., Stice S.L. (2017). Induced pluripotent stem cell-derived neural stem cell therapy enhances recovery in an ischemic stroke pig model. Sci. Rep..

[16] Yamashita T., Liu W., Matsumura Y., Miyagi R., Zhai Y., Kusaki M., Hishikawa N., Ohta Y., Kim S.M., Kwak T.H. (2017). Novel Therapeutic Transplantation of Induced Neural Stem Cells for Stroke. Cell Transplant..

[17] Boese A.C., Le Q.S.E., Pham D., Hamblin M.H., Lee J.P. (2018). Neural stem cell therapy for subacute and chronic ischemic stroke. Stem Cell Res. Ther..

[18] Takagi T., Yoshimura S., Sakuma R., Nakano-Doi A., Matsuyama T., Nakagomi T. (2017). Novel regenerative therapies based on regionally induced multipotent stem cells in post-stroke brains: Their origin, characterization, and perspective. Transl. Stroke Res..

[19] Eckert M.A., Vu Q., Xie K., Yu J., Liao W., Cramer S.C., Zhao W. (2013). Evidence for high translational potential of mesenchymal stromal cell therapy to improve recovery from ischemic stroke. J. Cereb. Blood Flow Metab..

[20] Benedek I., Bucur O., Benedek T. (2014). Intracoronary infusion of mononuclear bone marrow-derived stem cells is associated with a lower plaque burden after four years. J. Atheroscler. Thromb..

[21] Miyamoto M., Nakamura K., Shichinohe H., Yamauchi T., Ito M., Saito H., Kawabori M., Osanai T., Sasaki T., Houkin K., Kuroda S. (2018). Human Recombinant Peptide Sponge Enables Novel, Less Invasive Cell Therapy for Ischemic Stroke. Stem Cells Int..

[22] Uchida H., Niizuma K., Kushida Y., Wakao S., Tominaga T., Borlongan C.V., Dezawa M. (2017). Human Muse cells reconstruct neuronal circuitry in subacute lacunar stroke model. Stroke.

[23] Takizawa S., Nagata E., Nakayama T., Masuda H., Asahara T. (2016). Recent progress in endothelial progenitor cell culture systems: Potential for stroke therapy. Neurol. Med. Chir..

[24] Zhao Y.H., Yuan B., Chen J., Feng D.H., Zhao B., Qin C., Chen Y.F. (2013). Endothelial progenitor cells: Therapeutic perspective for ischemic stroke. CNS Neurosci. Ther..

[25] Wankhade U.D., Shen M., Kolhe R., Fulzele S. (2016). Advances in Adipose-Derived Stem Cells Isolation, Characterization, and Application in Regenerative Tissue Engineering. Stem Cells Int..

[26] Geng W., Tang H., Luo S., Lv Y., Liang D., Kang X., Hing W. (2019). Exosomes from miRNA-126-modified ADSCs promotes functional recovery after stroke in rats by improving neurogenesis and suppressing microglia activation. Am. J. Transl. Res..

[27] Gong B., Dong Y., Jiang W., Shan Y., Zhou B., Li W. (2019). Intravenous Transplants of Human Adipose Derived Stem Cell Protect the Rat From Ischemia-Induced Damage. J. Stroke Cardiovasc. Dis..

[28] Asahara T., Masuda H., Takahashi T., Kalka C., Pastore C., Silver M., Kearne M., Magner M., Isner J.M. (1999). Bone marrow origin of endothelial progenitor cells responsible for postnatal vasculogenesis in physiological and pathological neovascularization. Circ. Res..

[29] Gyöngyösi M., Hemetsberger R., Posa A., Charwat S., Pavo N., Petnehazy O., Petrasi Z., Pavo I.J., Hemetsberger H., Benedek I. (2010). Hypoxia-inducible factor 1-alpha release after intracoronary versus intramyocardial stem cell therapy in myocardial infarction. J. Cardiovasc. Transl. Res..

[30] Hill J.M., Zalos G., Halcox J.P., Schenke W.H., Waclawiw M.A., Quyyumi A.A., Finkel T. (2003). Circulating endothelial progenitor cells, vascular function, and cardiovascular risk. N. Engl. J. Med..

[31] Zhang Z.G., Zhang L., Jiang Q., Chopp M. (2002). Bone marrow-derived endothelial progenitor cells participate in cerebral neovascularization after focal cerebral ischemia in the adult mouse. Circ. Res..

[32] Ghani U., Shuaib A., Salam A., Nasir A., Shuaib U., Jeerakathil T., Sher F., O’Rourke F., Nasser A.M., Schwindt B. (2005). Endothelial progenitor cells during cerebrovascular disease. Stroke.

[33] Li Y.F., Ren L.N., Guo G., Cannella L.A., Chernaya V., Samuel S., Liu S., Wang H., Yang X.F. (2015). Endothelial progenitor cells in ischemic stroke: An exploration from hypothesis to therapy. J. Hematol. Oncol..

[34] Chen J., Zhang Z.G., Li Y., Wang L., Xu Y.X., Gautam S.C., Lu M., Zhu Z., Chopp M. (2003). Intravenous administration of human bone marrow stromal cells induces angiogenesis in the ischemic boundary zone after stroke in rats. Circ. Res..

[35] Arenillas J.F., Sobrino T., Castillo J., Dávalos A. (2007). The role of angiogenesis in damage and recovery from ischemic stroke. Curr. Treat. Options Cardiovasc. Med..

[36] Wei L., Keogh C.L., Whitaker V.R., Theus M.H., Yu S.P. (2005). Angiogenesis and stem cell transplantation as potential treatments of cerebral ischemic stroke. Pathophysiology.

[37] Sun Y., Jin K., Xie L., Childs J., Mao X.O., Logvinova A., Greenberg D.A. (2003). VEGF-induced neuroprotection, neurogenesis, and angiogenesis after focal cerebral ischemia. J. Clin. Investig..

[38] Carmeliet P., Storkebaum E. (2002). Vascular and neuronal effects of VEGF in the nervous system: Implications for neurological disorders. Semin. Cell Dev. Biol..

[39] Ferrara N., Gerber H.P. (2001). The role of vascular endothelial growth factor in angiogenesis. Acta Haematol..

[40] Zhang Z.G., Zhang L., Jiang Q., Zhang R., Davies K., Powers C., van Bruggen N., Chopp M. (2002). VEGF enhances angiogenesis and promotes blood-brain barrier leakage in the ischemic brain. J. Clin. Investig..

[41] Weis S.M., Cheresh D.A. (2005). Pathophysiological consequences of VEGF-induced vascular permeability. Nature.

[42] Yano A., Shingo T., Takeuchi A., Yasuhara T., Kobayashi K., Takahashi K., Muraoka K., Matsui T., Miyoshi Y., Hamada H. (2005). Encapsulated vascular endothelial growth factor—secreting cell grafts have neuroprotective and angiogenic effects on focal cerebral ischemia. J. Neurosurg..

[43] Croll S.D., Goodman J.H., Scharfman H.E. (2004). Vascular Endothelial Growth Factor (VEGF) in Seizures. Adv. Exp. Med. Biol..

[44] Bates D.O., Harper S.J. (2002). Regulation of vascular permeability by vascular endothelial growth factors. Vasc. Pharmacol..

[45] Forstreuter F., Lucius R., Mentlein R. (2002). Vascular endothelial growth factor induces chemotaxis and proliferation of microglial cells. J. Neuroimmunol..

[46] Valable S., Montaner J., Bellail A., Berezowski V., Brillault J., Cecchelli R., Divoux D., MacKenzie E.T., Bernaudin M., Roussel S. (2005). VEGF-induced BBB permeability is associated with an MMP-9 activity increase in cerebral ischemia: Both effects decreased by Ang-1. J. Cereb. Blood Flow Metab..

[47] Zheng Y., Murakami M., Takahashi H., Yamauchi M., Kiba A., Yamaguchi S., Yabana N., Alitalo K., Shibuya M. (2006). Chimeric VEGF-ENZ7/PlGF promotes angiogenesis via VEGFR-2 without significant enhancement of vascular permeability and inflammation. Arterioscler. Thromb. Vasc. Biol..

[48] Gomez R., Gonzalez-Izquierdo M., Zimmermann R.C., Novella-Maestre E., Alonso-Muriel I., Sanchez-Criado J., Remohi J., Simon C., Pellicer A. (2006). Low-dose dopamine agonist administration blocks vascular endothelial growth factor (VEGF)-mediated vascular hyperpermeability without altering VEGF receptor 2-dependent luteal angiogenesis in a rat ovarian hyperstimulation model. Endocrinology.

[49] Klein R., Mahlberg N., Ohren M., Ladwig A., Neumaier B., Graf R., Hoehn M., Albrechtsen M., Rees S., Fink G.R. (2016). The neural cell adhesion molecule-derived (NCAM)-peptide FG loop (FGL) mobilizes endogenous neural stem cells and promotes endogenous regenerative capacity after stroke. J. Neuroimmune Pharmacol..

[50] Gertz K., Priller J., Kronenberg G., Fink K.B., Winter B., Schröck H., Ji S., Milosevic M., Harms C., Böhm M. (2006). Physical activity improves long-term stroke outcome via endothelial nitric oxide synthase–dependent augmentation of neovascularization and cerebral blood flow. Circ. Res..

[51] Laufs U., Werner N., Link A., Endres M., Wassmann S., Jürgens K., Miche E., Böhm M., Nickenig G. (2004). Physical training increases endothelial progenitor cells, inhibits neointima formation, and enhances angiogenesis. Circulation.

[52] Zhang F., Wu Y., Jia J. (2011). Exercise preconditioning and brain ischemic tolerance. Neuroscience.

[53] Endres M. (2005). Statins and stroke. J. Cereb. Blood Flow Metab..

[54] Chen J., Zhang Z.G., Li Y., Wang Y., Wang L., Jiang H., Zhang C., Lu M., Katakowski M., Feldkamp C.S. (2003). Statins induce angiogenesis, neurogenesis, and synaptogenesis after stroke. Ann. Neurol..

[55] Zhang R., Wang L., Zhang L., Chen J., Zhu Z., Zhang Z., Chopp M. (2003). Nitric oxide enhances angiogenesis via the synthesis of vascular endothelial growth factor and cGMP after stroke in the rat. Circ. Res..

[56] Chen J., Zhang C., Jiang H., Li Y., Zhang L., Robin A., Katakowski M., Lu M., Chopp M. (2005). Atorvastatin induction of VEGF and BDNF promotes brain plasticity after stroke in mice. J. Cereb. Blood Flow Metab..

[57] Zhu B., Zhang L., Alexeyev M., Alvarez D.F., Strada S.J., Stevens T. (2009). Type 5 phosphodiesterase expression is a critical determinant of the endothelial cell angiogenic phenotype. Am. J. Physiol. Lung Cell Mol. Physiol..

[58] Li L., Jiang Q., Zhang L., Ding G., Zhang Z.G., Li Q., Ewing J.R., Lu M., Panda S., Ledbetter K.A. (2007). Angiogenesis and improved cerebral blood flow in the ischemic boundary area detected by MRI after administration of sildenafil to rats with embolic stroke. Brain Res..

[59] Zhang L., Zhang Z., Zhang R.L., Cui Y., LaPointe M.C., Silver B., Chopp M. (2006). Tadalafil, a long-acting type 5 phosphodiesterase isoenzyme inhibitor, improves neurological functional recovery in a rat model of embolic stroke. Brain Res..

[60] Gao F., Sugita M., Nukui H. (2005). Phosphodiesterase 5 inhibitor, zaprinast, selectively increases cerebral blood flow in the ischemic penumbra in the rat brain. Neurol. Res..

[61] Bednar M.M. (2008). The role of sildenafil in the treatment of stroke. Curr. Opin. Investig. Drugs.

[62] Pistrosch F., Herbrig K., Oelschlaegel U., Richter S., Passauer J., Fischer S., Gross P. (2005). PPARγ-agonist rosiglitazone increases number and migratory activity of cultured endothelial progenitor cells. Atherosclerosis.

[63] Gensch C., Clever Y.P., Werner C., Hanhoun M., Böhm M., Laufs U. (2007). The PPAR-γ agonist pioglitazone increases neoangiogenesis and prevents apoptosis of endothelial progenitor cells. Atherosclerosis.

[64] Seifert H.A., Pennypacker K.R. (2014). Molecular and cellular immune responses to ischemic brain injury. Trans. Stroke Res..

[65] Stonesifer C., Corey S., Ghanekar S., Diamandis Z., Acosta S.A., Borlongan C.V. (2017). Stem cell therapy for abrogating stroke-induced neuroinflammation and relevant secondary cell death mechanisms. Prog. Neurobiol..

[66] Bang O.Y. (2016). Clinical trials of adult stem cell therapy in patients with ischemic stroke. J. Clin. Neurol..

[67] Gyöngyösi M., Hemetsberger R., Wolbank S., Pichler V., Kaun C., Posa A., Petrasi Z., Petnehazy Ö., Hofer-Warbinek R., de Martin R. (2011). Delayed recovery of myocardial blood flow after intracoronary stem cell administration. Stem Cell Rev..

[68] Kempermann G., Wiskott L., Gage F.H. (2004). Functional significance of adult neurogenesis. Curr. Opin. Neurobiol..

[69] Nagy Z., Nardai S. (2017). Cerebral ischemia/repefusion injury: From bench space to bedside. Brain Res. Bull..

[70] Sandu R.E., Balseanu A.T., Bogdan C., Slevin M., Petcu E., Popa-Wagner A. (2017). Stem cell therapies in preclinical models of stroke. Is the aged brain microenvironment refractory to cell therapy?. Exp. Gerontol..

[71] Iadecola C., Anrather J. (2011). The immunology of stroke: From mechanisms to translation. Nat. Med..

[72] Popa-Wagner A., Filfan M., Uzoni A., Pourgolafshan P., Buga A.M. (2015). Poststroke cell therapy of the aged brain. Neural Plast..

[73] Widimsky P., Hopkins L.N. (2015). Catheter-based interventions for acute ischaemic stroke. Eur. Heart J..

[74] Kikuchi K., Tanaka E., Murai Y., Tancharoen S. (2014). Clinical trials in acute ischemic stroke. CNS Drugs.

[75] Boltze J., Arnold A., Walczak P., Jolkkonen J., Cui L., Wagner D.C. (2015). The dark side of the force–constraints and complications of cell therapies for stroke. Front. Neurol..

[76] Marei H.E., Hasan A., Rizzi R., Althani A., Afifi N., Cenciarelli C., Caceci T., Shuaib A. (2018). Potential of stem cell-based therapy for ischemic stroke. Front. Neurol..

[77] Bang O.Y., Kim E.H., Cha J.M., Moon G.J. (2016). Adult stem cell therapy for stroke: Challenges and progress. J. Stroke.

[78] Rosado-de-Castro P.H., Schmidt F.R., Battistella V., Lopes de Souza S.A., Gutfilen B., Goldenberg R.C., Kasai-Brunswick T.H., Vairo L., Silva R.M., Wajnberg E. (2013). Biodistribution of bone marrow mononuclear cells after intra-arterial or intravenous transplantation in subacute stroke patients. Regen. Med..

[79] Moniche F., Gonzalez A., Gonzalez-Marcos J.R., Carmona M., Piñero P., Espigado I., Garcia-Solis D., Cayuela A., Montaner J., Boada C. (2012). Intra-arterial bone marrow mononuclear cells in ischemic stroke: A pilot clinical trial. Stroke.

[80] Prasad K., Sharma A., Garg A., Mohanty S., Bhatnagar S., Johri S., Singh K.K., Nair V., Sarkar R.S., Gorthi S.P. (2014). Intravenous autologous bone marrow mononuclear stem cell therapy for ischemic stroke: A multicentric, randomized trial. Stroke.

[81] Yozbatiran N., Der-Yeghiaian L., Cramer S.C. (2008). A standardized approach to performing the action research arm test. Neurorehabil. Neural Repair.

[82] Díez-Tejedor E., Gutiérrez-Fernández M., Martínez-Sánchez P., Rodríguez-Frutos B., Ruiz-Ares G., Lara M.L., Gimeno B.F. (2014). Reparative therapy for acute ischemic stroke with allogeneic mesenchymal stem cells from adipose tissue: A safety assessment: A phase II randomized, double-blind, placebo-controlled, single-center, pilot clinical trial. J. Stroke Cerebrovasc. Dis..

[83] Prasad K., Mohanty S., Bhatia R., Srivastava M.V.P., Garg A., Srivastava A., Goyal V., Tripathi M., Kumar A., Bal C. (2012). Autologous intravenous bone marrow mononuclear cell therapy for patients with subacute ischaemic stroke: A pilot study. Indian J. Med. Res..

[84] Hess D.C., Wechsler L.R., Clark W.M., Savitz S.I., Ford G.A., Chiu D., Yavagal D.R., Uchino K., Liebeskind D.S., Auchus A.P. (2017). Safety and efficacy of multipotent adult progenitor cells in acute ischaemic stroke (MASTERS): A randomised, double-blind, placebo-controlled, phase 2 trial. Lancet Neurol..

[85] Steinberg G.K., Kondziolka D., Wechsler L.R., Lunsford L.D., Kim A.S., Johnson J.N., Bates D., Poggio G., Case C., McGrogan M. (2018). Two-year safety and clinical outcomes in chronic ischemic stroke patients after implantation of modified bone marrow–derived mesenchymal stem cells (SB623): A phase 1/2a study. J. Neurosurg..

[86] Mackie A.R., Losordo D.W. (2011). CD34-positive stem cells in the treatment of heart and vascular disease in human beings. Tex. Heart Inst. J..

[87] Clinicaltrials.gov Intravenous Stem Cells After Ischemic Stroke (ISIS). https://clinicaltrials.gov/ct2/show/NCT00875654?term=nct00875654&rank=1.

[88] Moniche F., Escudero I., Zapata-Arriaza E., Usero-Ruiz M., Prieto-León M., de la Torre J., Gamero M.A., Tamayo J.A., Ochoa-Sepúlveda J.J., Maestre J. (2015). Intra-arterial bone marrow mononuclear cells (BM-MNCs) transplantation in acute ischemic stroke (IBIS trial): protocol of a phase II, randomized, dose-finding, controlled multicenter trial. Int. J. Stroke.

[89] Banerjee S., Bentley P., Hamady M., Marley S., Davis J., Shlebak A., Nicholls J., Williamson D.A., Jensen S.L., Gordon M. (2014). Intra-arterial immunoselected CD34+ stem cells for acute ischemic stroke. Stem Cells Transl. Med..

[90] Clinicaltrials.gov Study of Autologous Stem Cell Transplantation for Patients With Ischemic Stroke. https://clinicaltrials.gov/ct2/show/NCT00473057?term=nct00473057&rank=1.

[91] Jin K., Sun Y., Xie L., Mao X.O., Childs J., Peel A., Logvinova A., Banwait S., Greenberg D.A. (2005). Comparison of ischemia-directed migration of neural precursor cells after intrastriatal, intraventricular, or intravenous transplantation in the rat. Neurobiol. Dis..

[92] Binder D.K., Rau G., Starr P.A. (2003). Hemorrhagic complications of microelectrode-guided deep brain stimulation. Stereotact. Funct. Neurosurg..

[93] Potts M.B., Silvestrini M.T., Lim D.A. (2013). Devices for cell transplantation into the central nervous system: Design considerations and emerging technologies. Surg. Neurol. Int..

[94] Chen L., Xi H., Huang H., Zhang F., Liu Y., Chen D., Xiao J. (2013). Multiple cell transplantation based on an intraparenchymal approach for patients with chronic phase stroke. Cell Transplant..

[95] Li G., Bonamici N., Dey M., Lesniak M.S., Balyasnikova I.V. (2018). Intranasal delivery of stem cell-based therapies for the treatment of brain malignancies. Expert Opin. Drug Deliv..

[96] Galeano C., Qiu Z., Mishra A., Farnsworth S.L., Hemmi J.J., Moreira A., Edenhoffer P., Hornsby P.J. (2018). The Route by Which Intranasally Delivered Stem Cells Enter the Central Nervous System. Cell Transplant..

[97] Al Fauzi A., Suroto N.S., Bajamal A.H., Machfoed M.H. (2016). Intraventricular transplantation of autologous bone marrow mesenchymal stem cells via Ommaya reservoir in persistent vegetative state patients after haemorrhagic stroke: Report of two cases & review of the literature. J. Stem Cells Regen. Med..

[98] Al Fauzi A., Sumorejo P., Suroto N.S., Parenrengi M.A., Wahyuhadi J., Turchan A., Mahyudin F., Suroto H., Rantam F.A., Machfoed M.H. (2017). Clinical Outcomes of Repeated Intraventricular Transplantation of Autologous Bone Marrow Mesenchymal Stem Cells in Chronic Haemorrhagic Stroke. A One-Year Follow Up. Open Neurol. J..

[99] Misra V., Lal A., El Khoury R., Chen P.R., Savitz S.I. (2011). Intra-arterial delivery of cell therapies for stroke. Stem Cells Dev..

[100] Savitz S.I., Misra V., Kasam M., Juneja H., Cox C.S., Alderman S., Aisiku I., Kar S., Gee AGrotta J.C. (2011). Intravenous autologous bone marrow mononuclear cells for ischemic stroke. Ann. Neurol..

[101] Battistella V., de Freitas G.R., da Fonseca L.M.B., Mercante D., Gutfilen B., Goldenberg R.C., Dias J.V., Kasai-Brunswick T.H., Wajnberg E., Rosado-de-Castro P.H. (2011). Safety of autologous bone marrow mononuclear cell transplantation in patients with nonacute ischemic stroke. Regen. Med..

[102] Kamiya N., Ueda M., Igarashi H., Nishiyama Y., Suda S., Inaba T., Katayama Y. (2008). Intra-arterial transplantation of bone marrow mononuclear cells immediately after reperfusion decreases brain injury after focal ischemia in rats. Life Sci..

[103] Li Y., Chopp M., Chen J., Wang L., Gautam S.C., Xu Y.X., Zhang Z. (2000). Intrastriatal transplantation of bone marrow nonhematopoietic cells improves functional recovery after stroke in adult mice. J. Cereb. Blood Flow Metab..

[104] Ge J., Guo L., Wang S., Zhang Y., Cai T., Zhao R.C., Wu Y. (2014). The size of mesenchymal stem cells is a significant cause of vascular obstructions and stroke. Stem Cell Rev..

[105] Schrepfer S., Deuse T., Reichenspurner H., Fischbein M.P., Robbins R.C., Pelletier M.P. (2007). Stem cell transplantation: The lung barrier. Transplant. Proc..

[106] Toma C., Wagner W.R., Bowry S., Schwartz A., Villanueva F. (2009). Fate of culture-expanded mesenchymal stem cells in the microvasculature: In vivo observations of cell kinetics. Circ. Res..

[107] Janowski M., Lyczek A., Engels C., Xu J., Lukomska B., Bulte J.W., Walczak P. (2013). Cell size and velocity of injection are major determinants of the safety of intracarotid stem cell transplantation. J. Cereb. Blood Flow Metab..

[108] Jung J.W., Kwon M., Choi J.C., Shin J.W., Park I.W., Choi B.W., Kim J.Y. (2013). Familial occurrence of pulmonary embolism after intravenous, adipose tissue-derived stem cell therapy. Yonsei Med. J..

[109] Kenmuir C.L., Wechsler L.R. (2017). Update on cell therapy for stroke. Stroke Vasc. Neurol..

[110] Seminatore C., Polentes J., Ellman D., Kozubenko N., Itier V., Tine S., Tritschler L., Brenot M., Guidou E., Blondeau J. (2010). The postischemic environment differentially impacts teratoma or tumor formation after transplantation of human embryonic stem cell-derived neural progenitors. Stroke.

[111] Chang D.J., Oh S.H., Lee N., Choi C., Jeon I., Kim H.S., Shin D.A., Lee S.E., Kim D., Song J. (2013). Contralaterally transplanted human embryonic stem cell-derived neural precursor cells (ENStem-A) migrate and improve brain functions in stroke-damaged rats. Exp. Mol. Med..

[112] Steward O., Sharp K.G., Yee K.M., Hatch M.N., Bonner J.F. (2014). Characterization of ectopic colonies that form in widespread areas of the nervous system with neural stem cell transplants into the site of a severe spinal cord injury. J. Neurosci..

[113] Bladin C.F., Alexandrov A.V., Bellavance A., Bornstein N., Chambers B., Coté R., Lebrun L., Pirisi A., Norris J.W. (2000). Seizures after stroke: A prospective multicenter study. Arch. Neurol..

[114] Bachier C., Potter J., Potter G., Sugay R., Shaughnessy P., Chan K., Jude V., Madden R., LeMaistre C.F. (2012). High white blood cell concentration in the peripheral blood stem cell product can induce seizures during infusion of autologous peripheral blood stem cells. Biol. Blood Marrow Transplant..

[115] Wichterle H., Lieberam I., Porter J.A., Jessell T.M. (2002). Directed differentiation of embryonic stem cells into motor neurons. Cell.

[116] Nagai N., Kawao N., Okada K., Okumoto K., Teramura T., Ueshima S., Umemura K., Matsuo O. (2010). Systemic transplantation of embryonic stem cells accelerates brain lesion decrease and angiogenesis. Neuroreport.

[117] Toda H., Takahashi J., Iwakami N., Kimura T., Hoki S., Mozumi-Kitamura K., Ono S., Hashimoto N. (2001). Grafting neural stem cells improved the impaired spatial recognition in ischemic rats. Neurosci. Lett..

[118] Guzman R., Bliss T., De Los Angeles A., Moseley M., Palmer T., Steinberg G. (2008). Neural progenitor cells transplanted into the uninjured brain undergo targeted migration after stroke onset. J. Neurosci. Res..

[119] Honmou O., Onodera R., Sasaki M., Waxman S.G., Kocsis J.D. (2012). Mesenchymal stem cells: Therapeutic outlook for stroke. Trends Mol. Med..

[120] Huang W., Mo X., Qin C., Zheng J., Liang Z., Zhang C. (2013). Transplantation of differentiated bone marrow stromal cells promotes motor functional recovery in rats with stroke. Neurol. Res..

[121] Lee J.S., Hong J.M., Moon G.J., Lee P.H., Ahn Y.H., Bang O.Y. (2010). A long-term follow-up study of intravenous autologous mesenchymal stem cell transplantation in patients with ischemic stroke. Stem Cells.

[122] Jiang J., Wang Y., Liu B., Chen X., Zhang S. (2018). Challenges and research progress of the use of mesenchymal stem cells in the treatment of ischemic stroke. Brain Dev..

[123] Abe K., Yamashita T., Takizawa S., Kuroda S., Kinouchi H., Kawahara N. (2012). Stem cell therapy for cerebral ischemia: From basic science to clinical applications. J. Cereb. Blood Flow Metab..

[124] Chen S.J., Chang C.M., Tsai S.K., Chang Y.L., Chou S.J., Huang S.S., Tai L.K., Chen Y.C., Ku H.H., Li H.Y. (2010). Functional improvement of focal cerebral ischemia injury by subdural transplantation of induced pluripotent stem cells with fibrin glue. Stem Cells Dev..

[125] Chang D.J., Lee N., Park I.H., Choi C., Jeon I., Kwon J., Oh S.H., Shin D.A., Do J.T., Lee D.R. (2013). Therapeutic potential of human induced pluripotent stem cells in experimental stroke. Cell Transplant..

[126] Ryu B., Sekine H., Homma J., Kobayashi T., Kawanata T., Shimizu T. (2019). Allogeneic adipose-derived mesenchymal stem cell sheet that produce neurological improvement with angiogenesis and neurogenesis in a rat stroke model. J. Neurosurg..

[127] Mu J., Bakreen A., Juntunen M., Korhonen P., Oinonen E., Cui L., Myllyniemi M., Zhao S., Miettinen S., Jolkkonen J. (2019). Combined Adipose Tissue-Derived Mesenchymal Stem Cell Therapy and Rehabilitation in Experimental Stroke. Front. Neurol..

[128] Clinicaltrials.gov Study of Modified Stem Cells (SB623) in Patients with Chronic Motor Deficit from Ischemic Stroke (ACTIsSIMA). https://clinicaltrials.gov/ct2/show/NCT02448641?term=nct02448641&rank=1.

[129] Clinicaltrials.gov Investigation of Neural Stem Cells in Ischemic Stroke (PISCES III). https://clinicaltrials.gov/ct2/show/NCT03629275?term=nct03629275&rank=1.

[130] Glass J.D., Boulis N.M., Johe K., Rutkove S.B., Federici T., Polak M., Kelly C., Feldman E.L. (2012). Lumbar intraspinal injection of neural stem cells in patients with amyotrophic lateral sclerosis: Results of a phase I trial in 12 patients. Stem Cells.

[131] Osanai T., Houkin K., Uchiyama S., Minematsu K., Taguchi A., Terasaka S. (2018). Treatment evaluation of acute stroke for using in regenerative cell elements (TREASURE) trial: Rationale and design. Int. J. Stroke.

[132] Broderick J.P., Adeoye O., Elm J. (2017). Evolution of the modified Rankin scale and its use in future stroke trials. Stroke.

[133] Chen K.L., Chen C.T., Chou Y.T., Shih C.L., Koh C.L., Hsieh C.L. (2014). Is the long form of the Fugl-Meyer motor scale more responsive than the short form in patients with stroke?. Arch. Phys. Med. Rehabil..

[134] Lyden P. (2017). Using the national institutes of health stroke scale: A cautionary tale. Stroke.

[135] Buzas R., Rogobete A.F., Popovici S.E., Mateescu T., Hoinoiu T., Sorop V.B., Bratu T., Ticlea M., Popoiu C.M., Sandesc D. (2018). Nuclear Transcription Factor Kappa B (NF-кB) and Molecular Damage Mechanisms in Acute Cardiovascular Diseases. A Review. J. Cardiovasc. Emerg..

[136] Xing R., De Wilde D., McCann G., Ridwan Y., Schrauwen J.T.C., van der Steen A.F.W., Gijsen F.J.H., Van der Heiden K. (2016). Contrast-enhanced micro-CT imaging in murine carotid arteries: A new protocol for computing wall shear stress. Biomed. Eng. Online.

[137] Cibis M., Potters W.V., Gijsen F.J., Marquering H., VanBavel E., van der Steen A.F., Nederveen A.J., Wentzel J.J. (2014). Wall shear stress calculations based on 3D cine phase contrast MRI and computational fluid dynamics: A comparison study in healthy carotid arteries. NMR Biomed..

[138] Zhou H., Meng L., Zhou W., Xin L., Xia X., Li S., Zheng H., Niu L. (2017). Computational and experimental assessment of influences of hemodynamic shear stress on carotid plaque. Biomed. Eng. Online.

[139] Nyulas T., Marton E., Rus V.A., Rat N., Ratiu M., Benedek T., Benedek I. (2018). Morphological features and plaque composition in culprit atheromatous plaques of patients with acute coronary syndromes. J. Cardiovasc. Emerg..

[140] Cheng C., Tempel D., van Haperen R., van der Baan A., Grosveld F., Daemen M.J., Krams R., de Crom R. (2006). Atherosclerotic lesion size and vulnerability are determined by patterns of fluid shear stress. Circulation.

[141] Masatsugu K., Itoh H., Chun T.H., Saito T., Yamashita J., Doi K., Inoue M., Sawada N., Fukunaga Y., Sakaguchi S. (2003). Shear stress attenuates endothelin and endothelin-converting enzyme expression through oxidative stress. Regul. Pept..

[142] Lind L., Andersson J., Larsson A., Sandhagen B. (2009). Shear stress in the common carotid artery is related to both intima-media thickness and echogenecity. Clin. Hemorheol. Microcirc..

[143] Zhang B., Gu J., Qian M., Niu L., Ghista D. (2018). Study of correlation between wall shear stress and elasticity in atherosclerotic carotid arteries. Biomed. Eng. Online.

[144] De Wilde D., Trachet B., De Meyer G.R., Segers P. (2016). Shear stress metrics and their relation to atherosclerosis: An in vivo follow-up study in atherosclerotic mice. Ann. Biomed. Eng..

[145] Jeong S.K., Lee J.Y., Rosenson R.S. (2014). Association between ischemic stroke and vascular shear stress in the carotid artery. J. Clin. Neurol..

[146] Seneviratne A.N., Cole J.E., Goddard M.E., Park I., Mohri Z., Sansom S., Udalova I., Krams R., Monaco C. (2015). Low shear stress induces M1 macrophage polarization in murine thin-cap atherosclerotic plaques. J. Mol. Cell. Cardiol..

[147] Oshida S., Mori F., Sasaki M., Sato Y., Kobayshi M., Yoshida K., Fujiwara S., Ogasawara K. (2018). Wall shear stress and T1 contrast ratio are associated with embolic signals during carotid exposure in endarterectomy. Stroke.

[148] Pedrigi R.M., Mehta V.V., Bovens S.M., Mohri Z., Poulsen C.B., Gsell W., Tremoleda J.L., Towhidi L., de Silva R., Petretto E. (2016). Influence of shear stress magnitude and direction on atherosclerotic plaque composition. R. Soc. Open. Sci..

[149] Roger V.L., Go A.S., Lloyd-Jones D.M., Benjamin E.J., Berry J.D., Borden W.B., Bravata D.M., Dai S., Ford E.S., Fox C.S. (2012). Heart disease and stroke statistics—2012 update: A report from the American Heart Association. Circulation.

[150] Bartosh T.J., Ylöstalo J.H., Mohammadipoor A., Bazhanov N., Coble K., Claypool K., Lee R.H., Choi H. (2010). Aggregation of human mesenchymal stromal cells (MSCs) into 3D spheroids enhances their antiin¬flammatory properties. Proc. Natl. Acad. Sci. USA.

[151] Frith J.E., Thomson B., Genever P.G. (2010). Dynamic three-dimensional culture methods enhance mesenchymal stem cell properties and increase therapeutic potential. Tissue Eng. Part C Methods.

[152] Sart S., Tsai A.C., Li Y., Ma T. (2013). Three-dimensional aggregates of mesenchymal stem cells: Cellular mechanisms, biological properties, and applications. Tissue Eng. Part B Rev..

[153] Imitola J., Park K.I., Teng Y.D., Nisim S., Lachyankar M., Ourednik J., Mueller F.J., Yiou R., Atala A., Sidman R.L. (2004). Stem cells: Cross–talk and developmental programs. Philos Trans. R Soc. Lond. B Biol. Sci. USA.

[154] George P., Bliss T.M., Mehta S., Sun G., Steinberg G.K. (2015). Abstract T MP17: Electrically preconditioned neural stem cells improve stroke recovery. Stroke.

[155] Teng Y.D., Lavik E.B., Qu X., Park K.I., Ourednik J., Zurakowski D., Langer R., Snyder E.Y. (2002). Functional recovery following traumatic spinal cord injury mediated by a unique polymer scaffold seeded with neural stem cells. Proc. Natl. Acad. Sci. USA.

[156] Aref A., Horvath R., McColl J., Ramsden J.J. (2009). Optical monitoring of stem cell-substratum interactions. J. Biomed. Opt..

[157] Kirouac D.C., Zandstra P.W. (2008). The systematic production of cells for cell therapies. Cell Stem Cell.

[158] Bracci R., Maccaroni E., Cascinu S. (2013). Transient sunitinib resistance in gastrointestinal stromal tumors. N. Engl. J. Med..

[159] Vijayavenkataraman S., Yan W.C., Lu W.F., Wang C.H., Fuh J.Y.H. (2018). 3D bioprinting of tissues and organs for regenerative medicine. Adv. Drug. Deliv. Rev..

[160] Mironov V., Kasyanov V., Markwald R.R. (2008). Nanotechnology in vascular tissue engineering: From nanoscaffolding towards rapid vessel biofabrication. Trends Biotechnol..

[161] Derakhshanfar S., Mbeleck R., Xu K., Zhang X., Zhong W., Xing M. (2018). 3D bioprinting for biomedical devices and tissue engineering: A review of recent trends and advances. Bioact. Mater..

[162] Tricomi B.J., Dias A.D., Corr D.T. (2016). Stem cell bioprinting for applications in regenerative medicine. Ann. N. Y. Acad. Sci..

[163] Ozawa T., Mickle D.A., Weisel R.D., Koyama N., Ozawa S., Li R.K. (2002). Optimal biomaterial for creation of autologous cardiac grafts. Circulation.

[164] Jin K., Mao X., Xie L., Galvan V., Lai B., Wang Y., Gorostiza O., Wang X., Greenberg D.A. (2010). Transplantation of human neural precursor cells in Matrigel scaffolding improves outcome from focal cerebral ischemia after delayed postischemic treatment in rats. J. Cereb. Blood Flow Metab..

[165] Boisserand L.S., Kodama T., Papassin J., Auzely R., Moisan A., Rome C., Detante O. (2016). Biomaterial Applications in Cell-Based Therapy in Experimental Stroke. Stem Cells Int..

[166] Delcroix G.J.R., Schiller P.C., Benoit J.P., Montero-Menei C.N. (2010). Adult cell therapy for brain neuronal damages and the role of tissue engineering. Biomaterials.

[167] Pakulska M.M., Ballios B.G., Shoichet M.S. (2012). Injectable hydrogels for central nervous system therapy. Biomed. Mater..

[168] Wang Y., Cooke M.J., Morshead C.M., Shoichet M.S. (2012). Hydrogel delivery of erythropoietin to the brain for endogenous stem cell stimulation after stroke injury. Biomaterials.

[169] Emerich D.F., Silva E., Ali O., Mooney D., Bell W., Yu S.J., Kaneko Y., Borlongan C. (2010). Injectable VEGF hydrogels produce near complete neurological and anatomical protection following cerebral ischemia in rats. Cell Transplant..

[170] Perale G., Rossi F., Sundstrom E., Bacchiega S., Masi M., Forloni G., Veglianese P. (2011). Hydrogels in spinal cord injury repair strategies. ACS Chem. Neurosci..

[171] Mdzinarishvili A., Sutariya V., Talasila P.K., Geldenhuys W.J., Sadana P. (2013). Engineering triiodothyronine (T3) nanoparticle for use in ischemic brain stroke. Drug Deliv. Transl. Res..

[172] Kalladka D., Muir K.W. (2014). Brain repair: Cell therapy in stroke. Stem Cells Cloning.

[173] Bifari F., Pacelli L., Krampera M. (2010). Immunological properties of embryonic and adult stem cells. World J. Stem Cells.

[174] Szajer J., Ho-Shon K. (2018). A comparison of 4D flow MRI-derived wall shear stress with computational fluid dynamics methods for intracranial aneurysms and carotid bifurcations—a review. Magn. Reson. Imaging.

[175] Szilágyi S.M., Popovici M.M., Szilágyi L. (2017). Automatic Segmentation Techniques of the Coronary Artery Using CT Images in Acute Coronary Syndromes. J. Cardiovasc. Emerg..

